# Direct MALDI-TOF MS and Antimicrobial Susceptibility Testing of Positive Blood Cultures Using the FAST^TM^ System and FAST-PBC Prep Cartridges—Performance Evaluation in a Clinical Microbiology Laboratory Serving High-Risk Patients

**DOI:** 10.3390/microorganisms10102076

**Published:** 2022-10-20

**Authors:** Khay Ugaban, Pil Pak, Rosemary C. She

**Affiliations:** 1 Keck Medical Center, University of Southern California, Los Angeles, CA 90033, USA; 2 Department of Pathology, Keck School of Medicine, University of Southern California, Los Angeles, CA 90033, USA

**Keywords:** bloodstream infection, rapid diagnostics, microorganism identification

## Abstract

Bloodstream infections are a leading cause of morbidity and mortality. The rapid diagnostic testing of positive blood cultures (PBCs) shortens times to effective therapy and the de-escalation of broad-spectrum empiric therapy. This is the first study examining the Qvella FAST^TM^ System for the rapid (~20 min) purification of microorganisms directly from PBCs using BacT/Alert^®^ FA/FAN bottles in the bioMérieux Virtuo instrument. We compared the performance of the FAST^TM^ System Liquid Colony^TM^ (LC), for immediate downstream ID and phenotypic AST, to standard workflow involving colonies obtained by overnight subculture. The LC yielded a concordant species ID by VITEK MS in 121/138 (87.7%) samples, identifying 32 different Gram-positive and Gram-negative species with 3/123 (2.6%) discordances. Compared to standard workflow, direct AST of the LC using VITEK^®^ 2 yielded 98.4% categorical agreement and 98.0% essential agreement. Very major error, major error, and minor error rates were 1.0%, 0.0%, and 1.8%, respectively, for Gram-negative organisms; and 1.9%, 0.2%, and 1.2%, respectively, for Gram-positive organisms. The median times from positive blood culture flag to results by FAST^TM^ System for ID and AST were 7.8 h and 15.7 h, respectively, versus 22.4 h and 36.6 h for standard workflow, respectively. In conclusion, the FAST^TM^ System provides reliable results for direct ID and AST from PBCs with significantly decreased turnaround times.

## 1. Introduction

Sepsis and antimicrobial resistance are two of our greatest public health challenges. There are more than 49 million cases of sepsis per year worldwide associated with approximately 11 million avoidable deaths per year [1]. Patients with bloodstream infection (BSI) due to antimicrobial-resistant organisms have a significantly higher mortality rates and costs of care [2]. The incidence of BSI due to multi-drug-resistant organisms has furthermore been increasing in recent decades [3]. The Centers for Disease Control and Prevention reports that ≥2 million people acquire an antibiotic-resistant infection each year in the United States (U.S.), of whom 23,000 people die [4]. In the U.S. alone, at least USD 62 billion is spent on sepsis [5]. Keys to improved outcomes in sepsis are effective antimicrobial therapy and antimicrobial stewardship. However, current blood culture technology requires several days for phenotypic antimicrobial susceptibility test (AST) results for bloodstream pathogens. There is an urgent need for diagnostic solutions that provide rapid and accurate identification and phenotypic AST results directly from positive blood cultures.

Many approaches have been developed to expedite results from positive blood cultures (PBCs). Direct species identification (ID) and detection of antimicrobial resistance genes using platforms providing panels of molecular markers has been found to shorten times to microbiological diagnosis and to effective antimicrobial therapy in BSI and to improve clinical outcomes [6]. These molecular approaches take 1–3 h to produce results and add substantially to laboratory costs as they are not replacements for the standard laboratory workflow. Many bloodstream pathogens and antimicrobial resistance genes are not covered in these panels. Given the ongoing emergence of novel antimicrobial resistance mechanisms, phenotypic AST approaches remain essential components of clinical microbiology laboratory operations to facilitate management of BSI, particularly de-escalation of antimicrobial therapy. A number of alternatives to subculturing PBC for ID and phenotypic AST have been developed, including the European Committee on AST (EUCAST) rapid AST method, various manual laboratory developed methods for cleaning, washing and concentrating microorganisms directly from the PBC, and rapid phenotypic AST by morphokinetic analysis [7,8,9]. The former two approaches are difficult to incorporate into laboratory workflow as they typically require extended hands-on time, whereas the latter involves increased costs associated with an additional high-tech system and offers a limited panel of antibiotics for AST.

A novel approach, the FAST^TM^ System and FAST-PBC Prep cartridge, has been developed for isolating and concentrating bacteria directly from a PBC. The FAST^TM^ System is a closed, rapid and automated sample preparation system that removes blood, cellular, and culture media matrix components from the PBC, while maintaining microbial cell viability. The instrument is capable of processing one or two cartridges in 20 or 30 min, respectively, in one run. The resulting material is a concentrated microbial suspension or Liquid Colony^TM^ (LC). The LC is functionally similar to a bacterial suspension from a solid colony in that it can be used for downstream ID and AST applications, including widely used phenotypic AST approaches and select targeted immunochromatographic resistance marker assays [10]. The short 2 min hands-on time minimizes barriers to workflow adaptation. In this study, we examined the accuracy of ID and AST results with application of the FAST^TM^ System for blood culture work-up using bioMerieux instruments; BacT/Alert^®^ Virtuo continuous monitoring blood culture system, Vitek^®^ MS for ID, and Vitek 2 for AST (bioMerieux, Lyon, France), not previously examined in conjunction with the FAST^TM^ System [11]. Performance was established comparing results of ID and AST from the LC vs. overnight subcultures (standard workflow). We also examined the impact on time to microbiological diagnosis in a unique 24/7 clinical laboratory system that serves a tertiary care adult patient population at risk of antimicrobial-resistant bloodstream infection.

## 2. Materials and Methods

**Samples.** One-hundred and thirty-one blood culture bottles (BACT/Alert^®^ FA/FN, bioMerieux) incubated in the BACT/ALERT^®^ Virtuo^®^ instrument (bioMerieux) were enrolled in the study. Blood cultures were collected per the clinical standard operating procedure (SOP) between February and August 2021 at Keck Medical Center of the University of Southern California (USC; Los Angeles, CA, USA). Any first-time positive blood culture for a patient during this time-period that had a Gram stain demonstrating bacterial organisms was eligible for inclusion in the study. Exclusion criteria included PBCs that were not processed within 16 h after flagging positive, PBCs from postmortem samples, PBCs from patients already included in the study, specimens found to be polymicrobial at time of Gram stain or after subculture, and PBCs which showed no visible organisms on Gram staining. Samples were de-identified upon inclusion in the study. The study was approved by the Institutional Review Board of USC (#HS-20-00813). Twenty-three additional PBCs were included in the study by seeding blood culture bottles with selected organisms from the Centers for Disease Control and Prevention AR Bank. Seeding of bottles followed bioMérieux manufacturer’s recommendations for preparing contrived specimens. Briefly, a 0.5 McFarland (~10^8^ CFU/mL) suspension of each bacterial isolate was serially diluted to a final concentration of ~250 CFU/mL per FA and FAN bottle. Each bottle was also inoculated with 5 mL of donor packed red blood cells.

**Standard workflow.** All PBC samples were subcultured on Columbia 5% sheep blood agar, MacConkey, and chocolate agar plates (BD BBL, Becton, Dickinson, and Company, Franklin Lakes, NJ, USA), with the addition of CDC anaerobic plate (BD BBL) for anaerobic PBC bottles. After overnight growth, ID from of plate colonies was performed using the Vitek^®^ MS matrix-assisted laser desorption ionization time-of-flight (MALDI-ToF) system and AST via VITEK^®^ 2. In addition, at time of PBC Gram stain, the Verigene BC-GP or BC-GN (Luminex Corp., Austin, TX, USA) was performed on prospective samples per manufacturers’ instructions. 

**FAST^TM^ System.** PBCs were processed using the FAST system (Qvella, ON, Canada) per the manufacturer’s recommendations. Sample cartridges were stored at room temperature prior to use. Briefly, 5 mL of broth from a PBC was aspirated using a syringe and needle and transferred to a sterile polypropylene tube, from which 2 mL was transferred to the FAST PBC Prep Cartridge and processed in the FAST^TM^ System instrument. The hands-on time to set up a run was approximately 2 min with a run time of 24 min for one sample. The FAST^TM^ System isolates and concentrates bacteria present in the PBC by lysing the human cellular material and washing away any debris present in the sample. The resultant LC was transferred from the cartridge to a sterile microcentrifuge tube, at which time volume of the LC was measured. The LC was then used for downstream ID and AST as described below.

**Identification (ID).** Identification was performed in quadruplicate using the Vitek^®^ MS system v3.2 (bioMerieux) for the LC, and in duplicate for overnight subculture per laboratory protocol. MALDI-ToF ID using the LC was performed by spotting 1 µL of LC per well of the target plate followed by formic acid extraction, spectra acquisition, and results interpretation per manufacturer’s instructions. If any of the four spots resulted in a high (99.9%) confidence ID correlating with the PBC Gram stain, the ID was accepted. If less than high confidence ID was obtained, a second FAST PBC Prep Cartridge was run from the PBC and MALDI-ToF was repeated. If ID results from LC differed from standard workflow, the purity plate from the LC was tested the next day by MALDI-ToF. MALDI-ToF on overnight subculture colonies was performed using the direct method, and formic acid extraction only performed in the case of no ID or poor ID, per the laboratory SOP. Times from PBC flagging positive to ID were recorded for both the FAST and standard workflows.

**Antimicrobial Susceptibility Testing (AST).** The VITEK^®^ 2 system was used for AST testing of both the LC and the overnight subculture. The VITEK^®^ 2 GP67 card was used for Gram-positive bacteria and GN79 card was used for Gram-negative bacteria (Table S1). A 0.5 McFarland (McF) was prepared in 0.45NaCl using the LC or colonies from the overnight subculture. For the purposes of the study, AST was performed on all PBC isolates from both LC and overnight subculture. Purity plates using 5% sheep blood agar were prepared from both the LC McF preparation and overnight subculture McF suspension. The VITEK^®^ 2 Expert system was used to interpret VITEK^®^ 2 AST results. Minimum inhibitory concentration (MIC) breakpoints were based on Clinical Laboratory Standards Institute M100 2021 [12]. The Sensititre Gram-Negative GNX3F AST Plate (Thermo Fisher Scientific, Waltham, Massachusetts) was used for *Stenotrophomonas maltophilia* AST. ETEST (bioMerieux) was used for *Streptococcus* spp. and anaerobes, as per manufacturers’ instructions. AST results obtained using the LC were compared to those from overnight subculture as the reference standard by categorical agreement (CA) and essential agreement (EA). CA was calculated as the proportion of number of categorical matches among total number of results. EA was calculated as the proportion of agreement between LC and standard workflow that were within a 2-fold dilution, among total number of results. Very major errors (VME), Major Errors (ME) and Minor Errors (mE) percentages were calculated. VME was defined as the proportion of susceptible results obtained by LC method to resistant results obtained by standard workflow, whereas ME was defined as the proportion of resistant results obtained by the LC to susceptible results obtained by standard workflow. mE was calculated as the percentage of all results in which one system obtained an intermediate result while the other system obtained a susceptible or resistant result [13]

**Statistics.** The Student’s *t*-test was used to test for differences in time from blood culture positivity to MALDI-ToF ID and phenotypic AST results between the accelerated workflow involving the FAST^TM^ system and the standard workflow using overnight subcultures.

## 3. Results

### 3.1. Study Samples

**Figure 1:**
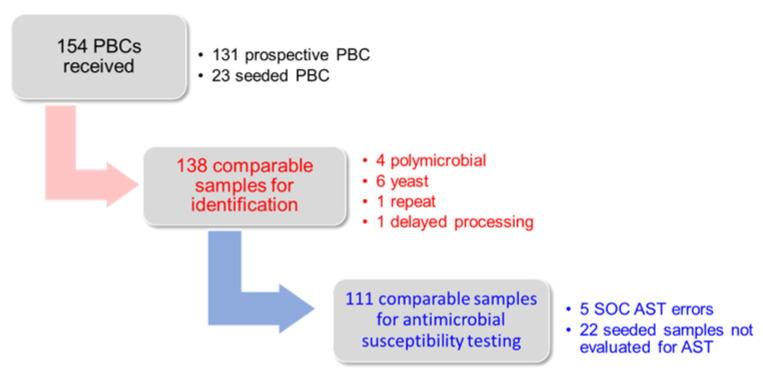
Study summary. During the study period, 131 positive blood cultures (PBCs) were received and 23 blood cultures were seeded with known organisms. Some samples were excluded for the reasons shown, leaving 138 samples for ID comparison and 111 samples for AST comparison.

A total of 154 PBC samples were initially included; 131 were prospective and 23 were seeded (Figure 1). Sixteen samples were excluded for the following reasons: polymicrobial culture (*n* = 4), yeast on Gram stain (*n* = 6), repeat culture from a study patient (*n* = 1), delayed processing on FAST^TM^ System (*n* = 1), FAST instrument error (*n* = 1), LC with visible blood (*n* = 2), no ID on standard workflow (*n* = 1). There was one specimen with a cartridge processing error initially but was successfully processed upon second a second cartridge from the PBC. Overall FAST^TM^ System processing success rate was 97.9% (139/142). The mean volume of the LC retrieved in successfully processed samples was 90 µL (median 80 µL). There was a total of 138 samples with comparable ID results: 22 seeded samples and 116 prospective samples from 59 male and 57 female patients (median age 60.0 y, interquartile range 50.0–67.3 y) and 22 seeded samples. Five additional prospective samples were excluded from AST comparison due to standard workflow errors. In addition, seeded samples were not run for AST, leaving 111 samples with comparable AST results (Table S2).

### 3.2. Identification Results

**Figure 2:**
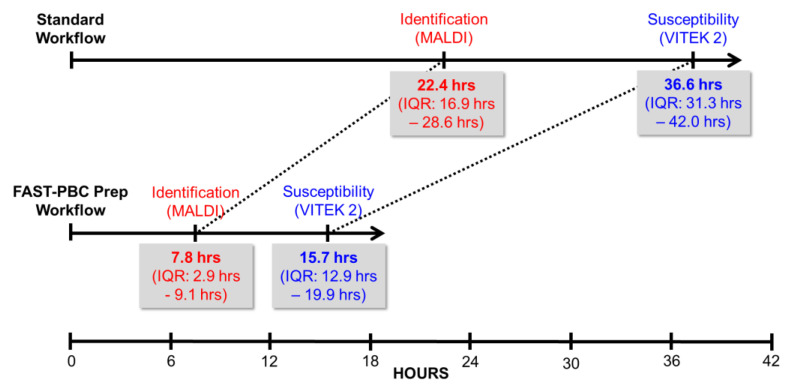
Timeline of microbiological procedures: median and interquartile range (IQR) of time from blood culture flagging positive to organism identification and phenotypic susceptibility results.

The most common species isolated in this study were *Staphylococcus epidermidis* (15.2%), *Escherichia coli* (13.8%), *Staphylococcus aureus* (12.3%), *Klebsiella* spp. (12.3%), and *Enterococcus* spp. (12.3%) (Table 1). Successful IDs obtained by VITEK^®^ MS of the LC included 16 different Gram-negative species (including 10 enteric species) and 16 different Gram-positive species (6 staphylococcal species, 5 streptococcal species, and 3 enterococcal species) (Table 1). Failure to produce IDs by VITEK^®^ MS using the LC was more common with Gram-positive species, especially streptococci, than with Gram-negative species. All but three positive IDs (97.4%) were concordant with IDs obtained with the standard workflow using overnight subcultures. One discordant sample was identified as a *S. epidermidis* by standard workflow and as *Listeria monocytogenes* by one of four spots of LC (99.9% confidence score). A second sample was identified as a *Stenotrophomonas maltophilia* using the overnight subculture colony and was identified as *Clostridioides difficile* (86.2% confidence) using the LC. A third sample was identified as *Streptococcus sanguinis* by standard workflow, while one of four spots from the LC yielded an identification by Vitek MS of *Mycobacterium genavense* (99.9% confidence). In each of these cases of discordant ID, only one of four spots produced a result by MALDI-ToF, PBC Gram stain did not correlate, and repeat MALDI-ToF ID performed on subcultures of the LC resulted in the same ID as that obtained with the standard workflow. Median time-to-result from when blood cultures flagged positive to MALDI-ToF ID was 7.8 h (interquartile range 2.9–9.1 h) versus 22.4 h (interquartile range 16.9–28.6 h) for the FAST^TM^ System versus the standard workflow, respectively (Figure 2). The difference in time to MALDI-ToF ID for the FAST^TM^ System versus the standard workflow was statistically significant (*p* < 10^−12^, Student’s *t*-test).

Table 2 compares the ID yield for the combination of LC plus VITEK MS vs. Verigene. The combination of LC plus VITEK MS gave correct species ID results in 88.2% and 83.1% of cases for Gram-negative and Gram-positive bacteria, respectively, for an overall success rate of 85.3%. By comparison, testing of PBCs with Verigene gave species ID results in 74.5% and 72.3% of cases of cases for Gram-negative and Gram-positive bacteria, respectively. Rates of no ID were similar for LC plus MALDI MS and Verigene.

### 3.3. Antimicrobial Susceptibility Testing Results

Median time-to-result from when blood cultures flagged positive to phenotypic AST was 15.7 h (interquartile range 12.9–19.9 h) versus 36.6 h (interquartile range 31.3–42.0 h) for the FAST^TM^ System versus the standard workflow, respectively (Figure 2). The difference in time to phenotypic AST for the FAST^TM^ System versus the standard workflow was statistically significant (*p* < 10^−8^, Student’s *t*-test).

A total of 613 AST results were compared from LC and standard workflow for Gram-negative bacteria, including 9 extended spectrum beta-lactamase and 1 carbapenem-resistant *Enterobacterales* organisms (Table 3). For Gram-negative bacteria, there was 1 VME (1.0%), 0 ME (0.0%) and 11 mE (1.8%), resulting in CA and EA of 98.0% and 98.4%, respectively, between the LC and standard workflow (Table S3). The single VME occurred with an *Enterobacter cloacae* isolate and ceftriaxone. Subgroup analysis within Gram-negative organism was carried out for *Enterobacterales* for which there were meaningful numbers for methods comparison (*n* = 39). CA and EA were 100% between LC and standard AST for the following drugs: amikacin, ampicillin, cefazolin, cefepime, ceftazidime, ertapenem.

Gentamicin, meropenem, piperacillin/tazobactam, tobramycin, and trimethoprim/sulfamethoxazole. Error rates for specific antimicrobial agents among *Enterobacterales* isolates: 4/31 mE for ampicillin/sulbactam, 1/39 mE for cefoxitin, 1/10 VME for ceftriaxone, and 1/39 mE for ciprofloxacin (Table 4). Six hundred and fifty-two AST results for Gram-positive bacteria, including four methicillin-resistant *S. aureus* organisms were analyzed. For Gram-positive bacteria, there were four VME (1.9%), one ME (0.2%) and eight mE (1.2%), resulting in CA and EA of 98.0% and 98.4%, respectively, between the LC and standard workflow (Table 3 and Table S3). All three VMEs for were found in three different cases of *S. epidermidis*: one each for clindamycin, erythromycin and trimethoprim/sulfamethoxazole. A fourth VME occurred with *Staphylococcus aureus* and penicillin involving a methicillin-susceptible organism. The error rates for these antimicrobials among staphylococci were 1/40 mE and 1/20 VME for clindamycin, 1/40 mE and 1/25 VME for erythromycin, 1/13 VME for trimethoprim/sulfamethoxazole, and 1/36 VME for penicillin. For the staphylococcal isolates evaluated, there was 100% CA and EA between the FAST-PBC Prep standard workflows for AST with the following agents: gentamicin, levofloxacin, linezolid, oxacillin, penicillin, quinupristin/dalfopristin, rifampin, tigecycline, vancomycin, and the cefoxitin screen. The only ME among Gram-positive bacteria involved an *E. faecium* isolate and tetracycline.

Among the 12 enterococcal isolates evaluated with 10 antimicrobials, there were 3 mEs and no VMEs. There were five viridans group streptococci that were evaluated via ETEST. LC compared to standard workflow resulted in 100% EA and CA for all drugs tested: penicillin, ceftriaxone, and vancomycin.

Anerobic isolates consisted of two Gram-negative and two Gram-positive organisms included in the respective calculations above. Comparison of LC and SOC AST for the four anaerobic isolates by ETEST showed 100% EA and CA for all drugs tested: ampicillin/sulbactam (*n* = 4), cefoxitin (*n* = 4), clindamycin (*n* = 2), penicillin (*n* = 4), and metronidazole (*n* = 4).

## 4. Discussion

The goal of this study was to evaluate the FAST^TM^ System for the rapid sample preparation of PBCs for same-day MALDI-ToF ID and phenotypic AST set up. Blood samples were collected in BacT/Alert FA/FAN bottles and incubated in the bioMerieux BacT/Alert Virtuo^®^ instrument, which has been found to have a shorter time to detection than other blood culture systems [14]. Direct testing of the LC obtained by the ~20 min processing of PBCs with the FAST^TM^ System was compared with standard workflow, yielding 98.4% concordance among samples subjected to MALDI-ToF ID by VITEK MS, and EA and CA of 98.4% and 98.0%, respectively, for AST by VITEK2. This is the first study to compare the accelerated FAST^TM^ System workflow with standard workflow using BacT/Alert FA/FAN bottles incubated in the Virtuo instrument and tested by VITEK MS.

In this study, 16 different Gram-negative species (including 10 enteric species) and 16 different Gram-positive species were identified from the LC using VITEK^®^ MS. This is a broader range of Gram-negative species than identified in previous evaluation of the FAST^TM^ System [11]. Of the samples that rendered an ID by the VITEK^®^ MS using the LC, 97.4% (120/123) were concordant with results using overnight subcultures. The LC from 10.8% of samples were not identified by VITEK MS, a result that was more common among Gram-positive than among Gram-negative organisms. In select cases where the PBC was processed a second time, the LC resulted in a positive VITEK MS result, which was an observation noted by others [11]. We observed infrequent errors in ID from the LC which may be further minimized by careful correlation of MALDI-ToF results with Gram stain. The overall rate of successful ID with the FAST^TM^ System workflow was comparable to that of Verigene (Table 2). The compatibility of the LC with MALDI presents several workflow advantages compared to molecular identification methods, including the ability to identify a broader range of organisms, shorter hands-on time, and faster turnaround time to ID and AST.

Phenotypic AST results are critical to appropriate management of patients with BSI, both in terms of ensuring effective antimicrobial therapy and for de-escalating therapy from broader initial empiric regimens. We found that inoculation of VITEK 2 AST cards with 0.5 McF suspensions of organisms with the LC obtained directly from PBCs using the FAST^TM^ System yielded AST results that were highly concordant with standard workflow results for ≥98.0% of bug-drug combinations tested. This is the first report comparing MICs obtained with the Etest^®^ for five streptococcal species and four anaerobe isolates, and no discordant results (100% EA and CA) were observed between the LC vs. colonies obtained from overnight subcultures. Though the numbers of streptococci and anaerobes tested were small, these data highlight the potential value of the LC used in combination with those susceptibility methods. In the study, only one VME with a Gram-negative organism was observed, involving the combination of *E. cloacae* with ceftriaxone. This scenario is well known to be associated with inducible AmpC beta-lactamase resistance [15] and this organism would not have been reported as ceftriaxone-susceptible using our laboratory’s AST reporting criteria. Of four VMEs observed with Gram-positive organisms, three involved *S. epidermidis* and the antibiotics clindamycin, trimethoprim-sulfamethoxazole, and erythromycin. These VMEs are unlikely to be clinically relevant, as these antibiotics are not widely used for the treatment of bloodstream infections with these organisms. A fourth VME involved *S. aureus* and penicillin. According to CLSI guidelines, any penicillin-susceptible *S. aureus* result that is negative with a nitrocefin-based test should be confirmed with the penicillin disk diffusion zone edge test prior to reporting [12].

Various factors could have contributed to the small number of discordant results in ID and AST between the FAST^TM^ system and standard workflow. In addition to the normal variance expected between methods, one factor that may have impacted AST results was the difference in organism sampling. The LC sample population reflects the organisms growing in the blood culture as a whole, whereas with the standard workflow, only a small number of colony-forming units on solid media is sampled for AST. Particularly if heteroresistance exists within the bacterial population, the two methods could produce differing results. Slight differences in ID or AST could also be related to variations in technique between operators, when operators were adjusting to a new method. Furthermore, the inadequate homogenization of the LC before application to the template for MALDI-ToF could have negatively affected ID results with the LC.

There are numerous methods to accelerate pathogen ID and AST direct from PBC. Based on our study experience, the advantages of the FAST^TM^ System were that it reliably purified microorganism cells from PBC broth in a short period of time with minimal labor. The rapid generation of an LC allowed for flexibility in downstream testing using existing laboratory instrumentation. The workflow of accelerating BSI work-up with the FAST^TM^ System would be similar for laboratories accustomed to using other rapid diagnostic platforms for BSI. A potential added benefit for the FAST^TM^ System approach is obviation of ID and AST from overnight subcultures, contingent on clinical validation studies to demonstrate that the rapid results are definitive and accurate. While in this study, the mean time to ID from time of PBC flag was over seven hours, this figure reflected inclusion of specimens that had flagged from the overnight shift and were processed by the morning shift personnel for the purposes of the study, as well as specimens which had to be repeat tested. Although we did not examine such approaches, targeted immunochromatographic or molecular antimicrobial resistance determinant testing could potentially be performed from the LC alongside phenotypic AST methods [16,17].

Our study results were generated from a pre-market version of the FAST^TM^ System; therefore, the actual performance of the commercially available device should be verified. While our study included a broad range of bacterial organisms, the sample size was somewhat limited. To address this limitation, we included coagulase-negative staphylococci in the AST comparisons even though most of the cases would normally be deemed as contaminants, not requiring AST work-up in clinical practice. Additional, focused testing of multidrug-resistant organisms is warranted in order to fully assess the AST performance characteristics including VME rates when using the LC from the FAST^TM^ System.

In summary, this is the first study reporting the performance of the FAST^TM^ System for same-day direct testing of PBCs obtained in BacT/Alert FA/FAN bottles and incubated in the Virtuo instrument. This study was also the first to compare rapid MALDI-ToF ID using VITEK MS for the LC vs. colonies obtained by overnight subculture. A high level of concordance was observed between ID and AST results obtained with the LC and standard workflow, including significant time savings versus the standard workflow, which would be expected to translate to reporting and optimization of antimicrobial therapy approximately one day sooner compared to the standard workflow. As this was a beta study, the FAST^TM^ System was not integrated into the standard workflow so time to result would theoretically be shorter once a laboratory has fully integrated the system into its standard of care. Two VMEs (*E. cloacae* and ceftriaxone and *S. aureus* and penicillin) could potentially have involved inducible resistance [15,18]. For this reason, it is important that laboratories using the FAST^TM^ System apply an Expert system and confirmatory testing as per CLSI guidelines, to prevent errors in reporting of antimicrobial susceptibility [19,20]. While our findings suggest that application of the LC to standard downstream ID and phenotypic AST methods is reliable, laboratories should continue to sub-culture samples to identify polymicrobial samples that were not apparent on initial Gram stain. Approaches such as the FAST^TM^ System that are cost effective, are easily integrated into the standard workflow, and dramatically shorten time to results are highly desirable for laboratories working to find methods and processes to improve care for patients with sepsis.

## Figures and Tables

**Table 1:** VITEK MS Results for Samples Included in Study by Species.

Gram-Negative	Prospective	Spiked	Total	No-ID	Discordant
*Escherichia coli*	18	1	19	2	0
*Klebsiella pneumoniae*	11	1	12	0	0
*Klebsiella aerogenes*	3	0	3	0	0
*Klebsiella oxytoca*	2	0	2	0	0
*Proteus mirabilis*	1	1	2	0	0
*Enterobacter cloacae*	3	1	4	0	0
*Serratia marcescens*	1	1	2	0	0
*Raoultella ornithinolytica*	0	1	1	0	0
*Morganella morganii*	0	1	1	0	0
*Citrobacter freundii*	0	1	1	0	0
*Pseudomonas aeruginosa*	6	5	11	1	0
*Stenotrophomonas maltophilia*	2	0	2	1	1
*Campylobacter fetus*	1	0	1	1	0
*Cardiobacterium hominis*	1	0	1	0	0
*Bacteroides fragilis*	1	0	1	0	0
*Bacteroides thetaiotaomicron*	1	0	1	0	0
**Subtotals**	**51**	**13**	**64**	**5**	**1**
**Gram Positive**	**Prospective**	**Spiked**	**Total**	**No-ID**	**Discordant**
*Staphylococcus epidermidis*	21	0	21	5	1
*Staphylococcus capitis*	3	0	3	1	0
*Staphylococcus caprae*	1	0	1	0	0
*Staphylococcus haemolyticus*	2	0	2	0	0
*Staphylococcus hominis*	3	0	3	0	0
*Staphylococcus aureus*	13	4	17	0	0
*Streptococcus anginosus*	1	0	1	1	0
*Streptococcus mitis/oralis*	3	0	3	2	0
*Streptococcus parasanguinis*	2	0	2	0	1
*Streptococcus vestibularis*	1	0	1	0	0
*Streptococcus pyogenes*	1	0	1	0	0
*Enterococcus faecalis*	7	2	9	0	0
*Enterococcus faecium*	5	2	7	0	0
*Enterococcus avium*	0	1	1	0	0
*Clostridium ramnosum*	1	0	1	0	0
*Clostridium tertium*	1	0	1	0	0
**Subtotals**	**65**	**9**	**74**	**9**	**2**
**Totals**	**116**	**22**	**138**	**14**	**3**

**Table 2:** Identification for Liquid Colony Plus MALDI MS vs. Verigene in prospective specimens.

	LC + MALDI MS			
	No ID	Discordant	Species ID	Total
Gram-negative	9.8% (5)	2.0% (1)	88.2% (45)	100% (51)
Gram-positive	13.8% (9)	3.1% (2)	83.1% (54)	100% (65)
Total	12.1% (14)	2.6% (3)	85.3% (99)	100% (116)
	**Verigene**			
	**No ID**	**Genus ID Only**	**Species ID**	**Total**
Gram-negative	13.7% (7)	11.8% (6)	74.5% (38)	100% (51)
Gram-positive	6.2% (4)	21.5% (14)	72.3% (47)	100% (65)
Total	9.5% (11)	17.2% (20)	73.3% (85)	100% (116)

**Table 3:** LC vs. standard workflow for AST using VITEK 2: summary of results for Gram-positive and Gram-negative isolates.

Gram-Positive AST Summary		
CA	639/652	98.0%
EA	642/652	98.4%
VME	4/210	1.9%
ME	1/431	0.2%
mE	8/652	1.2%
**Gram-Negative AST Summary**		
CA	601/613	98.0%
EA	603/613	98.4%
VME	1/96	1.0%
ME	0/506	0.0%
mE	11/613	1.8%

*Abbreviations:* CA, categorical agreement; EA, essential agreement, VME, very major error, ME, major error, mE, minor error.

**Table 4:** Comparison of AST results of LC vs. standard workflow: subgroup analysis for specimens containing *Enterobacterales*.

Antimicrobial	Essential Agreement	Categorical Agreement	Minor Errors	Major Errors	Very Major Errors
Amikacin	39/39 (100%)	39/39 (100%)	0/39	0/39	0/0
Ampicillin	30/31 (96.8%)	29/31 (93.5%)	2/31 (6.5%)	0/10	0/21
Ampicillin/Sulbactam	30/31 (96.8%)	27/31 (87.1%)	4/31 (12.9%)	0/19	0/10
Cefazolin	39/39 (100%)	36/39 (92.3%)	3/39 (7.8%)	0/22	0/17
Cefepime	39/39 (100%)	39/39 (100%)	0/39	0/36	0/1
Cefoxitin	38/39 (97.4%)	38/39 (97.4%)	1/39 (2.6%)	0/29	0/9
Ceftazidime	39/39 (100%)	39/39 (100%)	0/39	0/33	0/5
Ceftriaxone	38/39 (97.4%)	38/39 (97.4%)	0/39	0/29	1/10 (10.0%)
Ciprofloxacin	39/39 (100%)	39/39 (100%)	1/39	0/26	0/12
Ertapenem	39/39 (100%)	39/39 (100%)	0/39	0/38	0/1
Gentamicin	39/39 (100%)	39/39 (100%)	0/39	0/37	0/2
Meropenem	39/39 (100%)	39/39 (100%)	0/39	0/39	0/0
Piperacillin/Tazobactam	37/37 (100%)	37/37 (100%)	0/37	0/35	0/1
Tobramycin	39/39 (100%)	39/39 (100%)	0/39	0/35	0/1
Trimethoprim/Sulfamethoxazole	39/39 (100%)	39/39 (100%)	0/39	0/25	0/14
Total	558/567 (98.4%)	555/567 (97.9%)	11/567 (1.9%)	0/452	1/104 (1.0%)

## Data Availability

Data is contained within the article or Supplementary Materials.

## Supplementary Materials

The following supporting information can be downloaded at: https://www.mdpi.com/article/10.3390/microorganisms10102076/s1, Table S1: AST evaluation by antimicrobial agent and organism type; Table S2: All case study data; Table S3: Details of AST errors.
