# Supplementary material for: Direct MALDI-TOF MS and Antimicrobial Susceptibility Testing of Positive Blood Cultures Using the FASTTM System and FAST-PBC Prep Cartridges—Performance Evaluation in a Clinical Microbiology Laboratory Serving High-Risk Patients
Source: Microorganisms. 2022 Oct 20;10(10):2076. doi: 10.3390/microorganisms10102076 (PMC9612302; doi:10.3390/microorganisms10102076)
Supplement: Supplementary file 1 [file microorganisms-10-02076-s001.zip › Table S1.pdf]

Table S2: AST Evaluation of Antimicrobials by Organism Type

**Gram-negative**	***Enterobacterales***	***Pseudomonas* spp.**
Number of isolates	39	6
Amikacin	x	x
Ampicillin*	x	
Ampicillin/Sulbactam*	x	
Cefazolin	x	
Cefepime	x	x
Cefoxitin	x	
Ceftazidime	x	x
Ceftriaxone	x	
Ciprofloxacin	x	x
Ertapenem	x	
Gentamicin	x	x
Meropenem	x	x
Piperacillin/tazobactam	x	x
Tobramycin	x	x
Trimethoprim/sulfamethoxazole	x	x

*intrinsically resistant organisms not tested

**Gram-positive**	**Staphylococci**	**Enterococci**
Number of isolates	43	12
Ampicillin		x
Benzylpenicillin	x	x
Ciprofloxacin	x	x
Clindamycin	x	
Erythromycin	x	x
Gentamicin	x	
Levofloxacin	x	x
Linezolid	x	x
Moxifloxacin	x	
Oxacillin	x	
Quinupristin/Dalfopristin	x	x
Rifampicin	x	
Tetracycline	x	x
Tigecycline	x	x
Trimethoprim/sulfamethoxazole	x	
Vancomycin	x	x
