# Supplementary material for: Direct MALDI-TOF MS and Antimicrobial Susceptibility Testing of Positive Blood Cultures Using the FASTTM System and FAST-PBC Prep Cartridges—Performance Evaluation in a Clinical Microbiology Laboratory Serving High-Risk Patients
Source: Microorganisms. 2022 Oct 20;10(10):2076. doi: 10.3390/microorganisms10102076 (PMC9612302; doi:10.3390/microorganisms10102076)
Supplement: Supplementary file 1 [file microorganisms-10-02076-s001.zip › Table S3.pdf]

Table S3. Liquid Colony AST Errors compared to overnight culture with Vitek 2 AST

Species	Drug	LC Result MIC/Interpretation	SOC Result MIC/Interpretation	VME	ME	mE
**Gram-negative AST**						
Klebsiella pneumoniae	Ampicillin/Sulbactam	4 (S)	16 (I)			1
Klebsiella oxytoca	Ampicillin/Sulbactam	16 (I)	>=32 [R]			1
Klebsiella pneumoniae	Ampicillin/Sulbactam	16 (I)	>=32 [R]			1
Klebsiella pneumoniae	Ciprofloxacin	1 [R]	0.5 (I)			1
Klebsiella pneumoniae	ESBL Screen	NEG	POS			
Klebsiella pneumoniae	Ampicillin	16 (I)	>=32 [R]			1
Enterobacter cloacae	Ceftriaxone	≤1 (S)	4 (R)	1		
Klebsiella pneumoniae	Ampicillin/Sulbactam	16 (I)	>=32 [R]			1
Klebsiella aerogenes	Cefazolin	<=4 (I)	>=64 [R]			1
Klebsiella aerogenes	Cefazolin	<=4 (I)	>=64 [R]			1
E. coli	Cefoxitin	16 (I)	<=4 (S)			1
Klebsiella aerogenes	Cefazolin	<=4 (I)	>=64 [R]			1
Klebsiella pneumoniae	Ampicillin	16 (I)	>=32 [R]			1
**Species**	**Drug**	**LC Result**	**SOC result**	**VME**	**ME**	**mE**
**Gram-positive AST**						
S. hominis	Erythromycin	1 (I)	<=0.25 (S)			1
S. epidermidis	Clindamycin	0.5 (S)	2 (I)			1
S. haemolyticus	Moxifloxicin	1 (I)	2 R			1
S. aureus (MSSA)	Penicillin	0.06 (S)	0.25 (R)	1		
E. faecium	Linezolid	4 (I)	2 (S)			1
E. faecalis	Linezolid	4 (I)	2 (S)			1
S. epidermidis	Clindamycin	0.5 (S)	≥8 (R)	1		
S. epidermidis	Ciprofloxacin	2 (I)	4 (R)			1
S. epidermidis	Trimethoprim/Sulfamethoxazole	40 (S)	160 (R)	1		
S. epidermidis	Erythromycin	0.5 (S)	≥8 (R)	1		
E. faecalis	Erythromycin	4 (I)	>=8 [R]			1
E. faecium	Tetracycline	>=16 [R]	2 (S)		1	

Abbreviations:
LC = Liquid Colony
SOC = Standard of Care
VME = Very Major Error
ME = Major Error
mE = Minor Error
